# Surgical Approaches and Outcomes for 69 First Web Space Congenital Syndactyly Cases of the Hand

**DOI:** 10.1016/j.jhsg.2025.100832

**Published:** 2025-09-17

**Authors:** Manisha Banala, Sarah L. Struble, John R. Vaile, Apurva S. Shah, Shaun D. Mendenhall, Benjamin Chang

**Affiliations:** ∗ Perelman School of Medicine at the University of Pennsylvania, Philadelphia, PA; † Department of Plastic Surgery, University of California Irvine, Irvine, CA; ‡ Division of Plastic Surgery, Department of Surgery, Lehigh Valley Health Network, Allentown, PA; § Division of Orthopaedic Surgery, Children’s Hospital of Philadelphia, Philadelphia, PA; ‖ Division of Plastic & Reconstructive Surgery, Department of Surgery, Spencer Fox Eccles School of Medicine at the University of Utah, and Intermountain Primary Children’s Hospital, Salt Lake City, UT; ¶ Division of Plastic, Reconstructive, and Oral Surgery, Children’s Hospital of Philadelphia, Philadelphia, PA

**Keywords:** Congenital hand, First web space, Pediatric hand surgery, Syndactyly

## Abstract

**Purpose:**

First web space syndactyly presents a considerable reconstructive challenge, and because of the rarity and complexity of the condition, there is no consensus on the optimal approach to its reconstruction. This study therefore describes our experience with congenital first web space syndactyly, providing an overview of reconstructive techniques and outcomes.

**Methods:**

Sixty-nine cases (55 simple and 14 complex/complicated), from 2007 to 2022, were analyzed with descriptive statistics.

**Results:**

Dorsal commissural flaps were the initial operative approach in 100% of the complex/complicated cases, whereas Z-plasties were used in 64% of the simple cases. Full-thickness skin grafts were required in 12 (86%) of the complex/complicated cases and in 14 (26%) of the simple cases. Additional procedures such as amputation or osteotomy were performed in five complex/complicated (36%) and five simple (9.1%) cases. Range-of-motion deficits were the most common complication (26%), and 20 (29%) cases required revision surgery.

**Conclusions:**

Patients with first web syndactyly often require multiple operations and concurrent procedures on the index finger and thumb to optimize hand function. Reconstruction should be personalized to each patient’s unique clinical presentation.

**Type of study/level of evidence:**

Prognosis IIb.

Syndactyly, the second most common congenital hand anomaly after polydactyly, is characterized by the fusion of adjacent fingers.^1^, ^2^, ^3^ Simple syndactyly only involves soft tissue, whereas complex syndactyly also includes osseous or cartilaginous fusion. Complicated/complex syndactyly refers to accessory phalanges, tendons, muscles, or nerves interposed between fingers with a bony connection.^2^^,^^4^, ^5^, ^6^ Third web space syndactyly between the middle and ring finger is the most common (41%), followed by the fourth web space between the ring and small finger (27%).^4^ First web space syndactyly between the thumb and index finger is the rarest type (9.0%), is more commonly observed in syndromic patients such as those with Apert syndrome, and is often the most challenging to reconstruct.^5^^,^^7^ Although adequate thumb–index web space is critical for thumb and prehensile hand function, there is no clear consensus on the optimal approach to first web space syndactyly reconstruction.^8^

It is generally accepted that minor defects of the first web space can be corrected with Z-plasties, whereas more complex presentations require local or distant tissue transfers.^9^^,^^10^ However, previous studies describing the efficacy of various surgical approaches to first web space syndactyly reconstruction are limited by their small sample sizes. Additionally, recent work at our institution prompted further investigation by identifying first web syndactyly as an independent risk factor for revision surgery.^11^ This study therefore presents an overview of surgical techniques and outcomes at a major pediatric hospital over 15 years to treat congenital first web space syndactyly.

## Materials and Methods

With approval from the Institutional Review Board (21-019312), clinical information from January 2007 to January 2022 was extracted from a database of upper and lower extremity syndactyly cases at the Children's Hospital of Philadelphia, a tertiary, 627-bed academic pediatric hospital. For all photographs included in this article, informed consent for photographic release was obtained from the patient’s legal guardian. The analysis focused on the congenital, upper extremity, first web space syndactyly cases that were surgically reconstructed. Noncongenital, lower extremity, and nonoperable cases were excluded from the analysis. Patients with an absent thumb and/or first web space and patients with amniotic band syndrome were excluded.

Demographic and operative data were collected, and descriptive statistics were computed using statistical software. Demographic factors of interest included sex, age at the time of surgery, length of follow-up, laterality of syndactyly, and syndromic associations. Sex was defined as sex assigned at birth (male or female) as documented in the medical record; gender identity was not assessed. Operative data were recorded at the level of the individual web space, and factors on which data were collected included commissural flap design (eg, interdigitating triangular flap, rectangular flap, or Z-plasty), full-thickness skin graft (FTSG) use, and skin graft donor site. Additional procedures performed during the index operation, including tendon reconstructions, amputations, and osteotomies, were also recorded. Early postoperative complications occurring within 30 days of the initial reconstruction, including infection and dehiscence, along with late complications, such as pathologic scarring, range-of-motion deficits, web creep, angulation deformities, and flexion contracture, were reviewed. Details of revision surgeries, including indications and types of procedures, were noted.

## Results

The final analysis included 69 web space reconstructions in 56 patients. The cohort predominantly identified as White and non-Hispanic/Latino, with a 2.3:1 male-to-female ratio. The mean age at presentation was 1.1 years (range, 0–13.2 years), and the mean age at primary reconstruction was 2.9 years (range, 0.5–15.1 years). Patients followed up with their hand surgeon a mean of 2.7 years (SD, 3.4 years) after primary reconstruction (Table 1).

**Table 1:** Patient Demographics

Demographic Factor	Patients, n (%)
Total patients, n (%)	56 (100)
Sex, n (%)	
Male	39 (69.6)
Female	17 (30.4)
Race, n (%)	
White	39 (69.6)
Black	9 (16.1)
Asian	2 (3.6)
American Indian/Alaskan Native	1 (1.8)
Other	5 (8.9)
Ethnicity, n (%)	
Hispanic or Latino	2 (3.6)
Syndromic, n (%)	30 (53.6)
Age at presentation (y), mean (SD, range)	1.1 (2.9, 0–13.2)
Age at primary reconstruction (y), mean (SD, range)	2.9 (3.6, 0.5–15.1)
Follow-up (y), mean (SD, range)	2.7 (3.4, 0–13.2)
2019 ADI, mean (SD)∗	37.7 (23.7)

∗ The area deprivation index (ADI) was used as an indicator of the socioeconomic status of each patient based on home address and the corresponding nine-digit ZIP code. Higher values correlate with more socioeconomic disadvantages.

Of the 69 web spaces, 58% were associated with a syndrome (Table 2). Poland syndrome (n = 12 [17%]), Apert syndrome (n = 11 [16%]), and Greig cephalopolysyndactyly syndrome (n = 4 [5.8%]) were the most common syndromes reported. Most cases of first web space syndactyly were simple (n = 55 [80%]; Table 3).

**Table 2:** Web Spaces by Syndrome

Syndrome	Web Spaces, n (%)
Total web spaces	69 (100)
Symbrachydactyly (without Poland syndrome)	21 (30.4)
Synpolydactyly	7 (10.1)
Syndromic	40 (58.0)
Poland	12 (17.4)
Apert	11 (15.9)
Greig cephalopolysyndactyly	4 (5.8)
Split hand/split foot	3 (4.3)
Fraser	2 (2.9)
Syndrome suspected but not specified	2 (2.9)
Timothy	2 (2.9)
Arthrogryposis multiplex congenita	1 (1.4)
Pallister-Hall	1 (1.4)
VACTERL	1 (1.4)
Klippel-Feil	1 (1.4)

VACTERL, association of birth defects including vertebral, anal, cardiac, tracheoesophageal, renal, and limb anomalies.

**Table 3:** Web Space Classifications

Extent of Fusion	Simple	Complex/Complicated	Total
Incomplete	50	2	52
Complete	5	12	17
Total	55	14	

**Figure 1:**
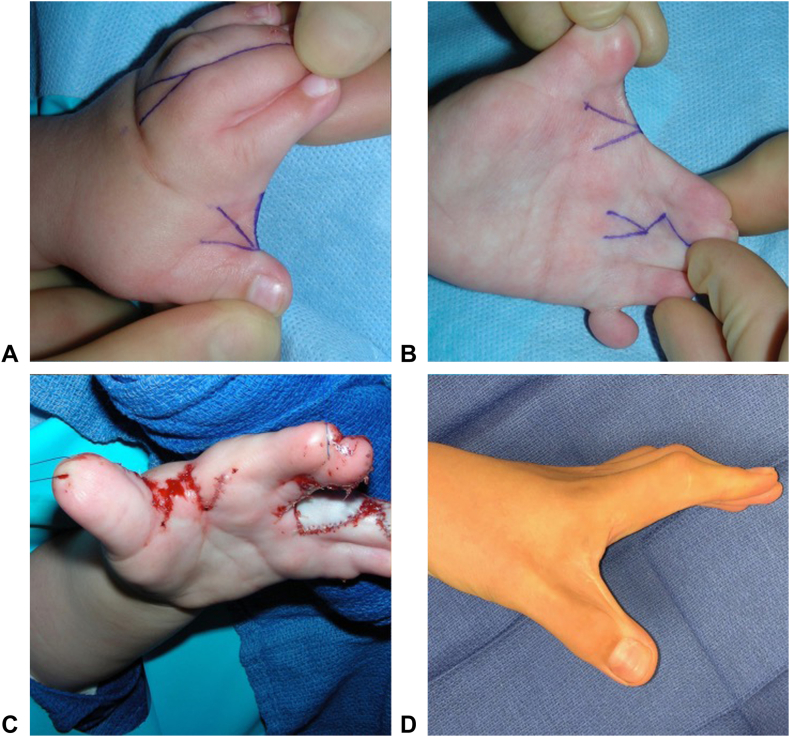
Example of a four-flap Z-plasty. **A** Dorsal preoperative markings. **B** Volar preoperative markings. **C** Result after closure. **D** Results at 14-year follow-up.

Z-plasties were performed as the initial surgery in 64% of the simple cases. Of these, 31% were four-flap Z-plasties (Fig. 1), 27% were classic, 3.6% were jumping man, and 1.8% were serial/compound (Table 4). The remaining cases were reconstructed using other local skin flaps.

**Table 4:** Initial Operative Approach to First Web Space Syndactyly

Operative Approach	Simple, n (%)	Complex/Complicated, n (%)
Total web spaces	55 (100)	14 (100)
Z-plasty	35 (63.6)	0
Four-flap	17 (30.9)	0
Classic	15 (27.3)	0
Jumping man	2 (3.6)	0
Serial/compound	1 (1.8)	0
Dorsal only commissural flap	15 (27.3)	8 (57.1)
Triangular	3 (5.5)	5 (35.7)
Rectangular/trapezoidal	9 (16.4)	2 (14.3)
Other	3 (5.5)	1 (7.1)
Dorsal and volar commissural flaps	4 (7.3)	6 (42.9)
Triangular	4 (7.3)	6 (42.9)
Other commissural flap	1 (1.8)	0
Snow-Littler	1 (1.8)	0
Skin graft	14 (25.5)	12 (85.7)
Hip/groin	9 (16.4)	6 (42.9)
Lower abdomen	2 (3.6)	4 (28.6)
Antecubital fossa	3 (5.5)	2 (14.3)
Additional procedures	5 (9.1)	5 (35.7)
Index ray amputation	0	1 (7.1)
Nonfunctional digit amputation	0	3 (21.4)
Thumb osteotomy	1 (1.8)	1 (7.1)
Tendon transfer (EIP to EPL)	2 (3.6)	0
Opponensplasty	1 (1.8)	0
Thumb polydactyly reconstruction	1 (1.8)	0

EIP, extensor indicis propius; EPL, extensor pollicis longus.

**Figure 2:**
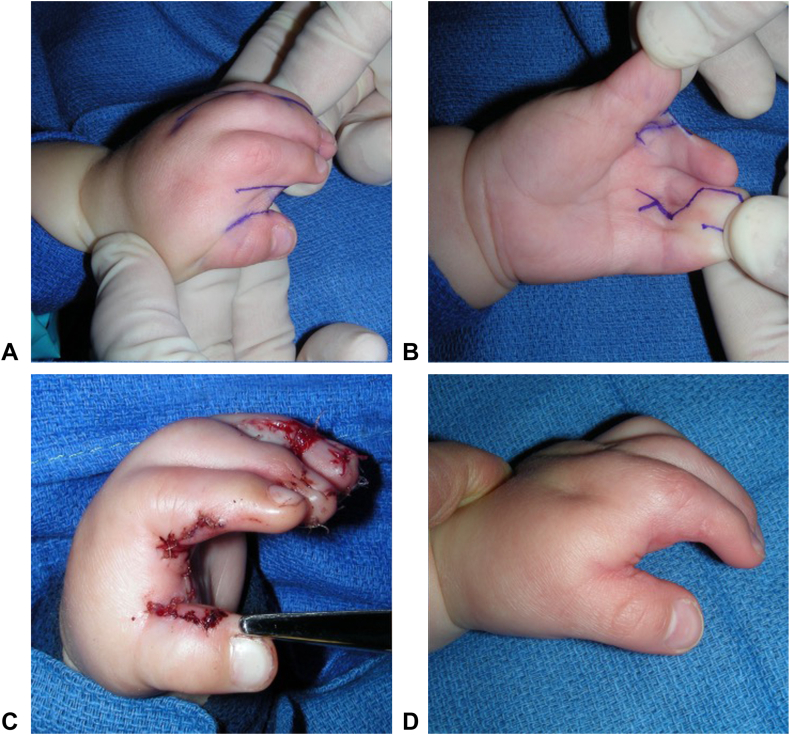
Example of a dorsal rectangular flap design. **A** Dorsal preoperative markings. **B** Volar preoperative markings. **C** Result after closure. **D** Results at 3-month follow-up.

**Figure 3:**
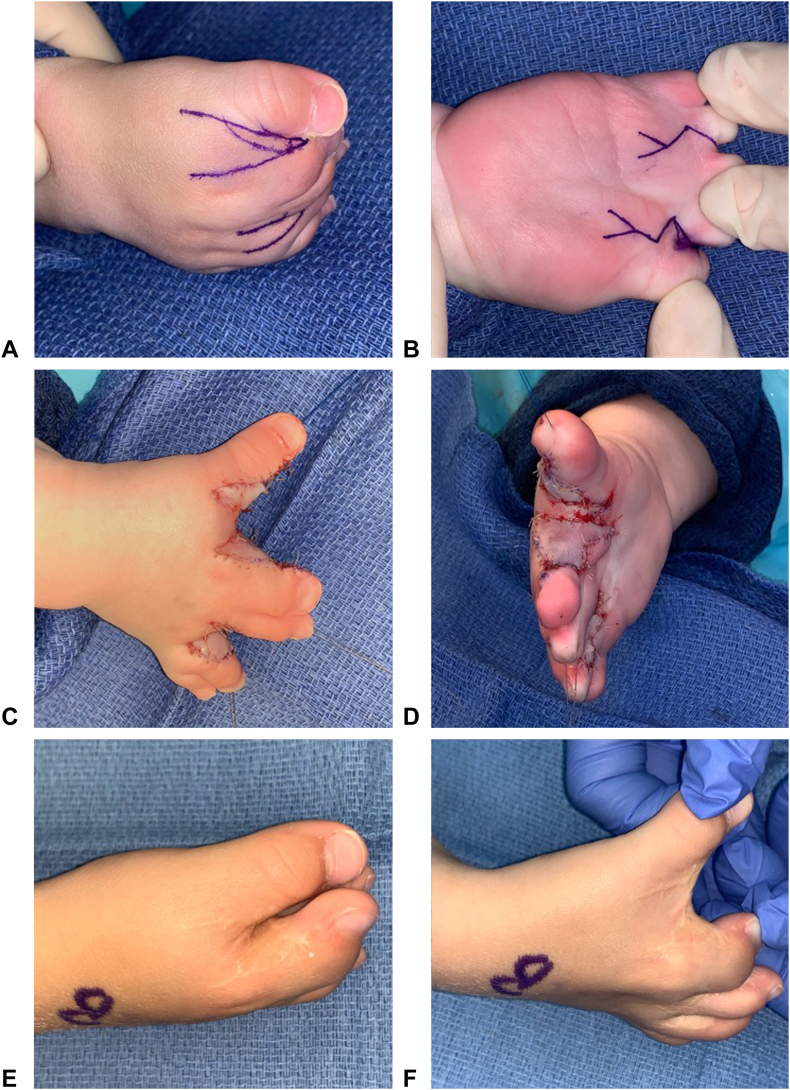
Example of dorsal and volar triangular flaps. **A** Dorsal preoperative markings. **B** Volar preoperative markings. **C, D** Result after closure. **E, F** Results at 23-month follow-up (note: patient later required revision surgery for web space deepening).

The most common commissural flap design used in the simple cases was a dorsal, proximally based rectangular flap (n = 9 [16%]; Fig. 2; Table 4). Triangular dorsal flaps were also commonly used, either alone (simple, n = 3 [5.5%]; complex/complicated, n = 5 [36%]) or in combination with a smaller volar triangular flap (simple, n=4 [7.3%]; complex/complicated, n = 6 [43%]; Fig. 3; Table 4). Supplementary skin coverage with FTSG was required in 14 (26%) simple cases and 12 (86%) complex cases, most commonly from the hip/groin and lower abdomen (Table 4). Additional procedures during the initial surgery, such as amputation and osteotomy, were performed in five complex (36%) and five simple (9.1%) cases (Table 4). For example, in four complex/complicated cases, amputation of the syndactylized index ray (n = 1) or nonfunctional/floating digits (n = 3) was necessary to create a functional first web space (Table 4).

Early complications occurring within 30 days of the initial reconstruction, such as infection, dehiscence, and cast changes, were infrequently observed (Table 5). However, late complications were recorded in most cases (simple, n = 34 [62%]; complex/complicated, n = 12 [86%]). Range-of-motion deficit was the most reported late complication; however, many of these cases had range-of-motion limitations before surgery. Web creep was also noted in both simple (n = 6 [11%]) and complex cases (n = 2 [14%]; Table 5).

**Table 5:** Complications After Initial Reconstruction

Complication Type	Simple (n = 55)	Complex/Complicated (n = 14)
Early complications, n (%)	3 (5.5)	3 (21.4)
Cast change required	3 (5.5)	2 (14.3)
Infection	0	0
Donor site dehiscence	0	1 (7.1)
Late complications, n (%)	34 (61.8)	12 (85.7)
Range-of-motion deficit	12 (21.8)	7 (50.0)
Hypertrophic scarring	3 (5.5)	2 (14.3)
Web creep	6 (10.9)	2 (14.3)
Web contracture	4 (7.3)	1 (7.1)
Thumb/IF angulation deformity	5 (9.1)	0
Thumb/IF flexion contracture	4 (7.3)	0

IF, index finger.

Twenty (29%) cases required at least one revision surgery, whereas six (8.7%) cases required two or more (Table 6). In most cases, normal growth of the affected hand narrowed the first web space, requiring either Z-plasty or FTSG (Table 6). Amputation of the index finger was also performed to widen the web space in four complex cases (Table 6). Other revisions addressed functional deficits or angulation. For example, wedge osteotomies were performed in three cases to correct radial angulation of the index finger, and thumb metacarpal rotational osteotomies were performed in two cases to enable opposition and tip-to-tip grasp.

**Table 6:** Revision Surgeries After First Web Space Syndactyly Reconstruction

Revision Surgery Type	Simple (n = 55)	Complex/Complicated (n = 14)
At least 1 revision surgery required, n (%)	12 (21.8)	8 (57.1)
Number of surgeries, median (range)	1 (1-3)	2 (1-4)
Revisions to deepen web space, n (%)		
Incision/release and FTSG	7 (12.7)	2 (14.3)
Local flap and FTSG	3 (5.5)	2 (14.3)
Z-plasty	5 (9.1)	0
Index ray amputation	0	4 (28.6)
Other revisional procedures, n (%)		
Index finger wedge osteotomy	3 (5.5)	0
Thumb metacarpal rotational osteotomy	1 (1.8)	1 (7.1)
Thumb proximal phalanx osteotomy, distraction, and bone graft	0	1 (7.1)
FPL lengthening and A1 pulley reconstruction	1 (1.8)	0

FPL, flexor pollicis longus.

Table 7 lists the procedures completed for patients, organized by the most common syndromes/diagnoses in the cohort. This table only includes procedures performed for initial web space creation and deepening.

**Table 7:** Procedures Performed for First Web Space Reconstruction by Syndrome/Diagnosis

Diagnosis	Procedures Performed
Symbrachydactyly (including Poland Syndrome) (n = 34)	Z-plasty or local flap and FTSG Modified Ghani (Dorsal rotational advancement flap) Index ray amputation Excision of remnant digits
Apert syndrome (n = 11)	
Upton type II	Z-plasty Index finger closing wedge osteotomy Fractional lengthening of the first dorsal interosseous and adductor pollicis
Upton type III	Dorsal triangular flaps and FTSG Index ray amputation
Ulnar longitudinal deficiency (n = 3)	Modified Ghani flap Dorsal triangular flap Release of the first dorsal interosseous from the thumb metacarpal
Hypoplastic thumb (n = 3)	Z-plasty Tendon transfer Opponensplasty
Cleft hand (n = 2)	Snow-Littler Index finger transposition
Radial longitudinal deficiency (n = 1)	Z-plasty Tendon transfer

**Figure 4:**
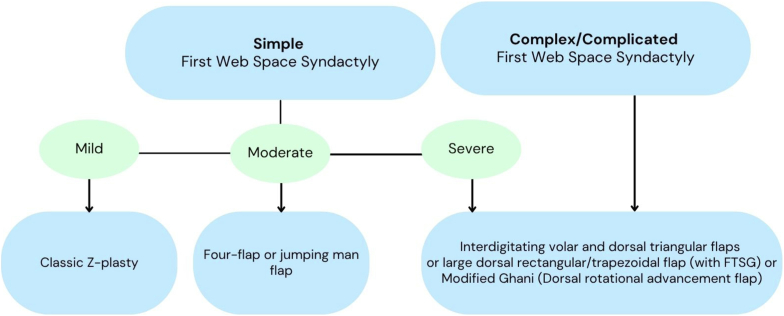
Decision tree to guide surgical planning for first web space syndactyly reconstruction.

With the experience aforementioned, the authors recommended using Figure 4 as a guide for surgical planning of first web space syndactyly reconstruction.

## Discussion

With one of the largest documented cohorts of congenital first web space syndactyly to date, this study provides a comprehensive overview of surgical approaches to its reconstruction.

Surgeon preferences for syndactyly repair vary depending on the web space affected and the complexity of the syndactyly. Repair of the second through fourth web spaces is often relatively straightforward, whereas the unique dimensions of the first web space make its reconstruction more complex. It is generally accepted that Z-plasties are sufficient for minor web space deficiency, whereas local or distant tissue recruitment is often required for more considerable syndactylization.^12^, ^13^, ^14^ However, various local flap designs have been described, and controversy over the best treatment approach persists.^15^ Flatt and Wood^16^ reported using multiple dorsal rotation flaps, whereas Friedman and Wood^17^ advocated for a single large dorsal transposition flap extending onto the dorsum of the index finger. For Apert syndrome specifically, Fearon^18^ described dorsal and volar triangular flaps of equal length and straight-line incisions to separate the fingers. For 16 patients with nonsyndromic congenital mitten hand and symbrachydactyly, Shen et al^19^ reported that a one-stage reconstruction, including a rotational osteotomy and dorsal interdigitating M flap, allowed opposition. This wide range of previously reported techniques prompted this overview of approaches to first web space syndactyly repair based on a large cohort of patients at our institution.

In the analysis of a large cohort (n = 77) of first web space deficiencies performed by Wu et al,^8^ including those with perinatal conditions such as cerebral palsy or neuromuscular disorders, first dorsal metacarpal artery pedicle flaps and Z-plasties were the most common operational techniques used, with 51% of cases requiring supplementary FTSG application. Although other complications were not described, 14% of the cohort required revision surgery.^8^ As previously mentioned, Friedman and Wood^17^ used dorsal transposition flaps and FTSG for 46 cases of congenital first web space contracture. Complications or unsatisfactory results, including inadequate correction, recurrent contracture, infection, and hypertrophic scarring, were documented in 37% of cases, and 26% of cases required revision surgery.^17^ Surgeons at our institution preferred Z-plasties for simple syndactyly and dorsal commissural flaps for complex and complicated syndactyly. Skin grafts were required in 38% of the 69 cases. We had an early complication rate of 8.7% when excluding preexisting range-of-motion deficits, and 39% of our cohort experienced late complications, with 29% requiring revision surgery. Our results are comparable with prior studies, indicating that no single strategy should be used for all presentations of syndactyly. Instead, the surgical approach should be personalized for each patient’s unique clinical presentation. Figure 4 can be used as a guide for surgical planning for first web space reconstruction.

**Figure 5:**
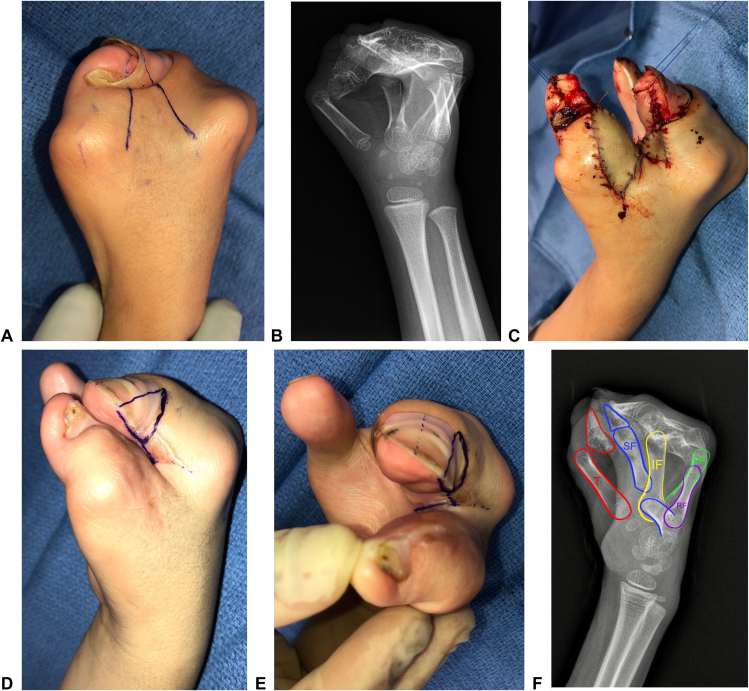

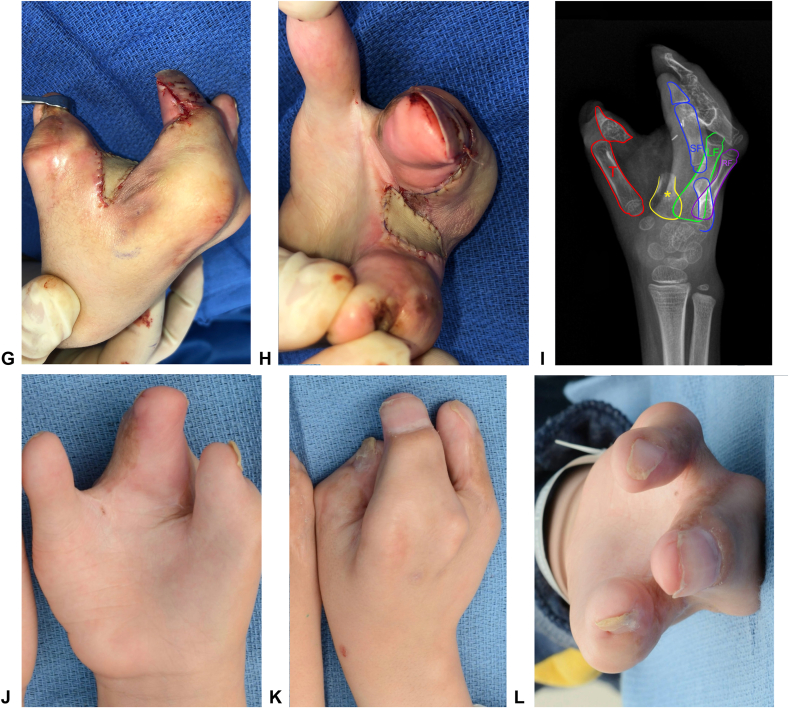
Example of a complex syndactyly case. The patient has Apert syndrome with Upton type three hands. They initially underwent first web space release with a dorsal trapezoidal flap and FTSG. However, the patient still had a tight first web space and limited active motion in the central three digits. Therefore, an index ray amputation was later performed to create a functional, deep first web space. **A** Preoperative clinical photo. **B** Preoperative x-ray. **C** Photo of the hand after the first stage release; note that the web space is still shallow. **D, E** Preoperative photos with surgical markings for the second stage surgery (index ray amputation). **F** X-ray of the hand after the first stage release. **G, H** Results after closure. **I** Postoperative x-rays. **J–L** Results at 4-month follow-up.

Owing to the diversity of clinical presentations and associated congenital anomalies, patients with first web space syndactyly often require multiple procedures on the affected hand. In the study by Wu et al,^8^ each patient required an average of 7.5 separate procedures, including joint stabilization procedures, extensor mechanism reconstructions, and osteotomies. In the study by Friedman and Wood,^17^ the most common additional procedures were Huber opposition transfer, ulnar collateral ligament reconstruction, and thumb metacarpal rotational osteotomy. In our cohort, 21% of complex/complicated syndactyly cases had a nonfunctional digit amputation, and 36% had an index ray amputation during the initial operation or subsequent revision. Although preservation of a five-digit hand is often prioritized, functional and anatomical limitations may necessitate finger amputation (Fig. 5). Other procedures besides amputation were also conducted during index or subsequent surgeries to address functional deficits. Thumb rotational osteotomies were performed to enable tip-to-tip pinch in cases where the thumb was coplanar with the other fingers. Angulation deformities of the index finger or thumb were corrected with osteotomies. Because of the multiple musculoskeletal abnormalities that often coincide with first web space syndactyly, patients and families should be informed that multiple procedures may be necessary to achieve a functional first web space. Additionally, in syndromic cases, multidisciplinary teams should try to coordinate procedures to limit anesthetic events.

This study had multiple limitations, including its retrospective nature. The data were also pooled from multiple surgeons, which may have introduced reporting bias into the complication rates. Additionally, the rarity of first web space syndactyly restricted the sample size to 69 cases in 56 patients. Although this is still one of the largest reported cohorts of congenital first web space syndactyly, it could not detect associations between syndactyly complexity or surgical approach and clinical outcomes. Furthermore, the cases analyzed in this study are from one center, and institutional traditions and training may have influenced the surgical techniques used. Finally, although all complications noted during follow-up visits were included in our analysis, patients may have had unreported dissatisfaction with the functional or cosmetic outcome of their procedure. Therefore, long-term data including patient-reported outcomes, web space functionality, and quality of life would add considerably to the literature.

## Conflicts of Interest

Dr Mendenhall is an educational consultant for PolyNovo, which does not relate to the content of this article. No benefits in any form have been received or will be received by the other authors related directly to this article.

## References

[1] Ahmed H., Akbari H., Emami A., Akbari M.R. (2017). Genetic overview of syndactyly and polydactyly. Plast Reconstr Surg Glob Open.

[2] Malik S. (2012). Syndactyly: phenotypes, genetics and current classification. Eur J Hum Genet.

[3] Savaci N., Hoŝnuter M., Tosun Z. (1999). Use of reverse triangular V-Y flaps to create a web space in syndactyly. Ann Plast Surg.

[4] Flatt A.E. (2005). Webbed fingers. Proc (Bayl Univ Med Cent).

[5] Braun T.L., Trost J.G., Pederson W.C. (2016). Syndactyly release. Semin Plast Surg.

[6] Geoghegan L., Knowles B.G., Nikkhah D. (2020). Syndactyly. J Surg Case Rep.

[7] Mahindroo S., Tabaie S. (2023). Syndactyly in the pediatric population: a review of the literature. Cureus.

[8] Wu T., Walchak A., Rayan G.M. (2021). Congenital thumb-index web space deficiency. Hand (N Y).

[9] Green D.P., Wolfe S.W. (2011).

[10] Mende K., Watson A., Stewart D.A. (2020). Surgical treatment and outcomes of syndactyly: a systematic review. J Hand Surg Asian Pac Vol.

[11] Belardo Z.E., Graham E.M., Pehnke M. (2024). Revision surgery following primary reconstruction for hand syndactyly. J Hand Surg Am.

[12] Adani R., Tarallo L., Marcoccio I., Fregni U. (2006). First web-space reconstruction by the anterolateral thigh flap. J Hand Surg Am.

[13] Coombs C.J., Mutimer K.L. (1994). Tissue expansion for the treatment of complete syndactyly of the first web. J Hand Surg Am.

[14] Hsu V.M., Smartt J.M.J., Chang B. (2010). The modified V-Y dorsal metacarpal flap for repair of syndactyly without skin graft. Plast Reconstr Surg.

[15] Foucher G., Medina J., Navarro R., Pajardi G. (2000). Value of a new first web space reconstruction in congenital hand deformities. A study of 54 patients. Chir Main.

[16] Flatt A.E., Wood V.E. (1970). Multiple dorsal rotation flaps from the hand for thumb web contractures. Plast Reconstr Surg.

[17] Friedman R., Wood V.E. (1997). The dorsal transposition flap for congenital contractures of the first web space: a 20-year experience. J Hand Surg Am.

[18] Fearon J.A. (2003). Treatment of the hands and feet in Apert syndrome: an evolution in management. Plast Reconstr Surg.

[19] Shen X.F., Yin F., Gasteratos K. (2022). Thumb and first webspace reconstruction in nonsyndromic congenital mitten hand with symbrachydactyly. J Plast Reconstr Aesthet Surg.

